# Dose-volume factors associated with ear disorders following intensity modulated radiotherapy in nasopharyngeal carcinoma

**DOI:** 10.1038/srep13525

**Published:** 2015-09-01

**Authors:** Ji-Jin Yao, Guan-Qun Zhou, Li Lin, Wang-Jian Zhang, Ying-Lin Peng, Lei Chen, Ling-Long Tang, Yan-Ping Mao, Jun Ma, Ying Sun

**Affiliations:** 1 Department of Radiation Oncology, Sun Yat-sen University Cancer Center, State Key Laboratory of Oncology in South China, Collaborative Innovation Center for Cancer Medicine, Guangzhou 510060, Guangdong Province, People’s Republic of China; 2 Department of Medical Statistics and Epidemiology & Health Information Research Center & Guangdong Key Laboratory of Medicine, School of Public Health, Sun Yat-sen University, Guangzhou 510080, Guangdong Province, China

## Abstract

This study is to identify significant dosimetric parameters for ear disorders in nasopharyngeal carcinoma (NPC) patients treated with intensity modulated therapy only. Ninety-seven patients with NPC were retrospectively reviewed. Organs at risk (OARs) in the auditory apparatus were contoured. Dose–volume histogram parameters were generated for the Eustachian tube (ET), tympanic cavity (TC), mastoid air cells, vestibular apparatus, cochlea and internal auditory canal (IAC). Ear disorders were rated 0 (none), 1 (mild) or 2 (severe) by a clinician blinded to radiation doses; Grade 2 ear disorders was the study end-point. Multivariate analysis revealed ET.D_30_ (dose to 30% of ET volume) >52.75 Gy and M.D_0.5CC_ (dose to 0.5 ml of mastoid volume) >41.04 Gy (*OR* = 3.77, *P* = 0.012 and *OR* = 1.27, *P* = 0.033, respectively) were associated with Grade 2 ear disorders. Our results demonstrated that post-irradiation ear disorders remain a common late toxicity in NPC after IMRT. ET.D_30_ and M.D_0.5CC_ should be considered during IMRT treatment plan optimization, review and approval.

Nasopharyngeal carcinoma (NPC) is common among Asian populations, especially in Southern China where the age-standardized incidence is 20–50 per 100,000^1^. Radical radiotherapy is the primary treatment modality for non-disseminated NPC due to its anatomic location and radio-sensitivity; however, radiotherapy for NPC is notoriously difficult due to the invasive characteristics of the tumor and its proximity to critical structures. Ear disorders induced by radiotherapy, characterized by ear pain, tinnitus, otitis media with effusion (OME), hearing loss and other symptoms, are one of the most important dose-limiting factors and a frequently observed complication in patients with NPC^2^,^3^. Previous studies demonstrated that 16–48% of patients with NPC undergoing two-dimensional radiotherapy (2D-CRT) developed persistent or recurrent post-irradiation ear disorders^4^,^5^,^6^,^7^.

Intensity modulated radiotherapy (IMRT) has gradually replaced 2D-CRT as the primary means of radiotherapy due to its superior tumor target coverage and normal tissue sparing. The design of appropriate dose constraints for the organs at risk (OARs) during optimization of IMRT treatment plans can enable significantly better OAR sparing and reduce subsequent complications^8^. However, the dose limits for many OARs, including the auditory apparatus, are poorly characterized. Furthermore, much existing data is based on the experience of clinicians in the 2D-CRT era and has a lack of solid clinical evidence^9^,^10^,^11^,^12^. There is a critical need for more accurate information regarding the dose limits for the auditory apparatus in patients with NPC receiving IMRT.

Therefore, we retrospectively reviewed a cohort of patients with NPC who did not suffer from any ear disorder before radiotherapy and analyzed dose–volume histogram (DVH) predictors for ear disorders. The aims of this study were to assess the incidence of ear disorders and determine the radiation dose constraints for the particular structures associated with severe ear disorders in patients with NPC treated with IMRT.

## Materials and Methods

### Study population

Between February 2009 and May 2010, 123 patients with newly-diagnosed, histologically-proven, non-distant metastatic NPC patients who received IMRT only were included in this study. Data from physical examinations, imaging and therapeutic schedules was obtained for all patients, along with information on any acute and late normal-tissue effects. Of the 123 patients, 26 patients were excluded due to unilateral or bilateral ear disorders before radiotherapy (ear pain, tinnitus, otitis media with effusion [OME], hearing loss or other symptoms); this study was based on the remaining 97 patients. The characteristics of the 97 patients with NPC are presented in Table 1. This retrospective study was approved by the Institutional Review Board of Sun Yat-sen University Cancer Center and in accord with the institutional policy to protect the patients’ private information. The need for informed consent was waived.

### Clinical staging and evaluation of ear disorders

All patients completed a pre-treatment evaluation that included a physical examination, nasopharyngeal and neck MRI, chest radiography, abdominal sonography, and whole body bone scan. Additionally, 7 patients (7.2%) underwent positron emission tomography computed tomography (PET-CT). Medical records and imaging studies were recorded, and all patients were restaged according to the seventh edition of the Union for International Cancer Control/American Joint Committee on Cancer (UICC/AJCC) staging system^13^.

Ear disorders were divided into three grades according to the criteria reported by Nishimura *et al.*^14^ as follows: Grade 0: no complaints or symptoms; Grade 1: ear pain, tinnitus, OME, hearing loss, or other symptoms not posing difficulty in daily life or requiring any treatment; Grade 2: ear pain, tinnitus, OME, hearing loss, or other symptoms posing difficulty in daily life and/or requiring treatment. In the current study, we defined ear disorders Grade 2 as the study endpoint.

### MRI protocol

MRI was performed using a 1.5-Tesla system (Signa CV/i; General Electric Healthcare, Chalfont St. Giles, United Kingdom), where a head-and-neck combined coil was used to examine the area spanning the suprasellar cistern to the inferior margin of the sternal end of the clavicle. T1-weighted fast spin-echo images in the axial, coronal and sagittal planes (repetition time, 500–600 ms; echo time, 10–20 ms) and T2-weighted fast spin-echo MRI in the axial plane (repetition time, 4000–6000 ms; echo time, 95–110 ms) were obtained before injection of contrast material. Following intravenous injection of gadopentetate dimeglumine (0.1 mmol/kg body weight Gd-DTPA, Magnevist; Bayer-Schering, Berlin, Germany), spin-echo T1-weighted axial and sagittal sequences and spin-echo T1-weighted fat-suppressed coronal sequences were performed sequentially. Diffusion-weighted MRI using line scan diffusion images was performed with a pelvic phased-array coil with *b*-values of 5 and 800 s/mm^2^. Section thickness was 5 mm with a 1 mm interslice gap for the axial plane, and 6 mm with a 1 mm interslice gap for the coronal and sagittal planes.

### Auditory apparatus contouring and data collection

**Figure 1:**
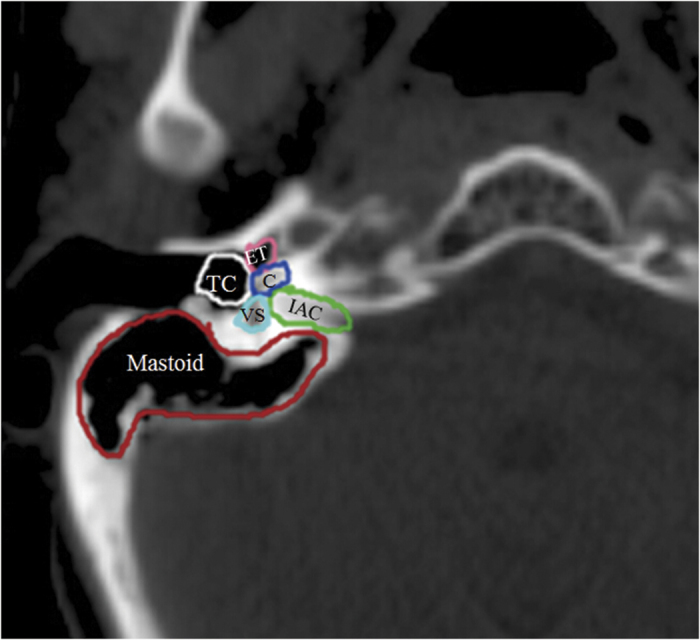
CT imaging of the anatomy of the Eustachian tube (ET), tympanic cavity (TC), mastoid air cells (Mastoid), vestibular apparatus (VS), cochlea (C), and internal auditory canal (IAC).

The volumes of each component of the auditory apparatus, including the Eustachian tube (ET), tympanic cavity (TC), mastoid air cells, vestibular apparatus (VS), cochlea and internal auditory canal (IAC), were contoured on every slice by a radiologist. Based on anatomic definitions, a reasonable contouring method for the auditory apparatus (Fig. 1) was employed^8^. We collected the DVH parameters for each component of the auditory apparatus, for example, the mean dose to the ET, volume of the ET, D_0.1CC_ (the dose to 0.1 ml of the ET volume), D_0.01CC_, D_0.02CC_, D_0.03CC_, D_0.04CC_, D_0.05CC_, D_0.06CC_, D_1_ (the dose to 1% of the ET volume), D_5_, D_10_, D_20_, D_30_, D_35_, D_40_, D_45_, D_50_, D_60_, V_10_ (the volume of the ET that received more than 10 Gy), V_20_, V_30_, V_35_, V_40_, V_45_, V_50_, V_55_, V_60_, V_65_, V_70_ and V_75_. A total of 152 DVH parameters were reviewed.

### Radiation therapy

Target volumes were delineated using our institutional treatment protocol in accordance with the International Commission on Radiation Units and Measurements reports 50 and 62.11 The prescribed doses were: a total dose of 68–70 Gy in 30–33 fractions at 2.13–2.27 Gy/fraction to the planning target volume (PTV) of the primary GTV, 60 Gy to the PTV of CTV-1 (i.e. high-risk regions), 54 Gy to the PTV of CTV-2 (i.e. low-risk regions and neck nodal regions), and 60–68 Gy to the nodal GTV in 30–33 fractions. Treatment was delivered once daily over five fractions per week.

### Follow up and statistical analysis

MRI examinations were performed before radiotherapy in all patients; all of the data was available for this analysis. Patients were followed-up at least every three months in the first three years and every six months thereafter. Routine follow-up care included a complete head and neck examination, hematology and biochemistry profiles, chest radiography and abdominal sonography. Follow-up neck and/or nasopharynx MRI was performed every 6–12 months, especially for cases with suspected tumor recurrence or RT-induced complications.

Associations between selected DVH parameters and the incidence of ear disorders were evaluated. Receiver operating characteristic (ROC) curve analysis was applied to assess the performance of putative predictors for the occurrence of severe ear disorders. The predictive value of each parameter was evaluated based on the area under the ROC curve (AUC). Predictors with an AUC significantly higher than 0.5 were then transformed into binary variables according to the optimal cut-off points selected and incorporated into univariate logistic regression models. Finally, a multivariate logistic regression model was used to identify predictors that have significant impacts on severe ear disorders. All analyses were performed using R3.1.2.

## Results

Overall, 97 patients (194 ears) met the inclusion criteria and were included in this study. The median follow-up time for this cohort was 43.5 months (range, 31.2 to 50.6 months) and the final follow-up was performed on January 15th, 2015. The 4-year overall survival rate was 96.9% and the local recurrence-free rate was 93.8%.

The incidence of ear disorders in the current study was 44.3% (43 of 97 patients), with 15.5% (15 patients) having bilateral ear disorders. When the ears were considered individually, the incidence of ear disorders was 30% (58/194) for the entire cohort. Based on the most recent examination, 70% (136/194) ears had Grade 0 ear disorders, 22% (42/194) had Grade 1 ear disorders, and 8% (16/194) had Grade 2 ear disorders.

### Univariate & multivariate analysis

A total of 152 DVH parameters (see materials and methods) were analyzed in the current study, including the mean dose and volume for each component of the auditory apparatus. Initially, ROC curves were generated to analyze the value of all these putative predictors for the occurrence of severe ear disorders. A total of 152 DVH parameters with an AUC >0.5 were incorporated into univariate analysis. According to the optimal cut-points from the ROC curves, these 152 parameters were transformed into binary variables. Univariate analysis showed that sixty dosimetric parameters were significantly associated with severe ear disorders (Table 2). The mean doses to the ET and mastoid were significantly associated with severe ear disorders (*P* = 0.033 and *P* = 0.016 respectively). On the other hand, we observed no association between the mean dose to the TC, VS, cochlea or IAC and severe ear disorders (all *P* > 0.05).

Multivariate analysis by forward elimination of insignificant explanatory variables was performed to identify risk factors associated with severe ear disorders; all of the significant DVH parameters from univariate analysis were incorporated into a multivariate logistic regression model. Multivariate analysis showed that only ET.D_30_ (OR = 3.77, *P* = 0.012) and M.D_0.5cc_ (OR = 1.27, *P* = 0.033) were associated with severe ear disorders (Table 3); other factors, including the doses to the TC, VS, cochlea and IAC were not significant.

### Subgroup analysis of independent DVH parameters for ear disorders

**Figure 2:**
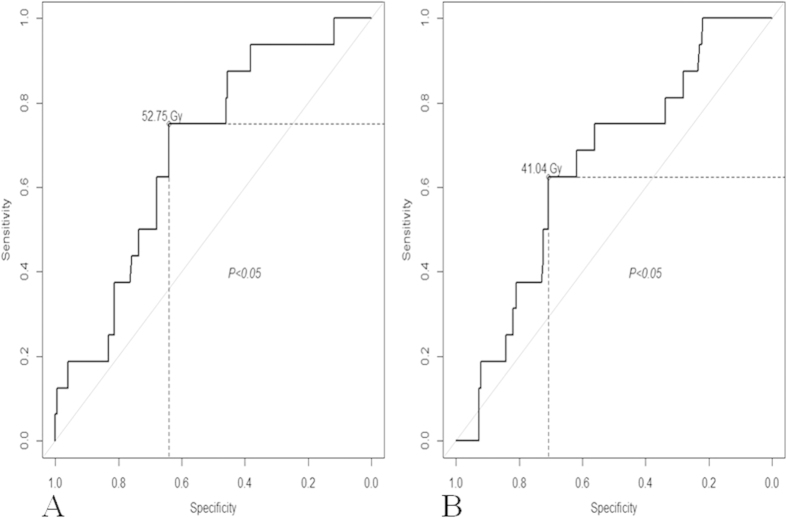
(**A**) Receiver operating characteristic (ROC) curve analysis for the ET.D_30_ (dose to 30% of the Eustachian tube volume). The cutoff point for the ET.D_30_ was determined as 52.75 Gy for patients with NPC treated with IMRT. (**B**) Receiver operating characteristic (ROC) curve for the M.D_0.5CC_ (dose to 0.5 ml of the mastoid volume). The cutoff point for the M.D_0.5CC_ was determined as 41.04 Gy for patients with NPC treated with IMRT.

The analysis above demonstrated that ET.D_30_ and M.D_0.5CC_ were associated with ear disorders. The performance of these predictors for ear disorders was further assessed using ROC curves. The area under the ROC curve was 0.69 for the ET.D_30_ (*P* = 0.012; Fig. 2A). As shown in Fig. 2A, it would be appropriate to consider an ET.D_30_ of 52.75 Gy as the dose limit for severe ear disorders (sensitivity, 0.63; specificity, 0.71). The mean dose limits of the ET.D_30_ associated with Grade 0 ear disorders, Grade 1 and Grade 2 ear disorders were 50.08 Gy ± 0.71 Gy, 54.74 ± 0.82 Gy and 55.38 Gy ± 0.73 Gy, respectively. Multiple comparisons revealed that the mean dose to the ET.D_30_ was significantly different between ears with Grade 0 and Grade 2 ear disorders (*P* = 0.041).

The area under the ROC curve was 0.65 for the M.D_0.5CC_ (*P* = 0.033; Fig. 2B). From Fig. 2B, it would be appropriate to consider a M.D_0.5CC_ of 41.04 Gy as the dose tolerance with respect to severe ear disorders (sensitivity, 0.63; specificity, 0.71). The mean doses to the M.D_0.5CC_ for Grade 0, Grade 1 and Grade 2 ear disorders were 41.70 Gy ± 0.58 Gy, 45.96 Gy ± 0.73 Gy and 45.32 Gy ± 0.79 Gy, respectively. Multiple comparisons revealed that the mean dose to the M.D_0.5CC_ was significantly different between ears with Grade 0 and Grade 2 ear disorders (*P* = 0.044).

The ET.D_30_ also had a significant association with ear disorders in patients with NPC treated with IMRT. In patients with an ET.D_30_ <52.75 Gy, the incidence of ear disorders was 17% (13/38) compared to 38% (45/119) in patients with an ET.D_30_ ≥ 52.75 Gy (*P* = 0.012). The M.D0.5_CC_ was also associated with the incidence of ear disorders: in the group with a M.D_0.5CC_ <41.04 Gy, the incidence of ear disorders was 21% (13/62) compared to 33% (45/145) in the group with a M.D_0.5CC_ ≥ 41.04 Gy (*P* = 0.033). Therefore, it is important to select the optimal DVH parameters to predict the occurrence of ear disorders.

## Disussion

### Incidence of radiation-induced ear disorders

Ear disorders are one of the most serious late sequelaes after radical radiotherapy in NPC and adversely affect the patient’s quality of life. In one analysis, 136 of 325 patients (41.8%) with head and neck tumors were found to have ear disorders after 2D-CRT^15^. With the introduction of IMRT, the incidence of ear disorders is expected to decrease. However, Zeng *et al.*^16^ and Liang *et al.*^17^ reported the incidence of radiation-induced ear disorders in patients with NPC treated using IMRT was 46.2% and 52.9%, respectively. We revealed that ear disorders persisted in approximately 44.3% (43/97) of patients with NPC treated with IMRT, which is roughly consistent with Zeng *et al.*^16^. Therefore, the application of IMRT has seemingly failed to reduce the occurrence of ear disorders in NPC.

The factors underlying this observation may be interpreted as follows. Although IMRT is superior in terms of escalated target dose coverage, the CTV inevitably includes the medial part of the ET and mastoid. It is well recognized that the ET is a critical portion of the auditory apparatus responsible for tubal dysfunction^18^, and direct radiation damage to the mastoid air cells can promote a non-infectious inflammatory response^19^. Therefore, the dose of radiation to the cartilaginous portion of the ET and mastoid is the same or even higher with IMRT compared to 2D-CRT, and the current dose limit for the auditory apparatus does little to reduce the incidence of radiation-induced ear disorders.

### Significant components of the auditory apparatus involved in ear disorders

In the current analysis, the ET.D_30_ was identified as the most valuable predictor of ear disorders, which implies that ET radiation-induced injury plays an important role in the occurrence of post-radiotherapy ear disorders. One reason underlying this observation may be that damage to the ET and surrounding structures (tensor veli palatini muscle, cartilage, nerves) leads to development of negative pressure in the ME, resulting in transudation of serous fluid. This fluid can impede sound conduction through the ME and become inoculated with bacteria from the nasopharynx^19^. The M.D_0.5CC_ was also relevant to the occurrence of Grade 2 ear disorders. Although the exact mechanisms associated with this observation remain unknown, one possible cause is that delivery of a high radiation dose to the mastoid air cells can lead to a noninfectious inflammatory response, resulting in transudation of serous fluid. Furthermore, mucosal damage within the mastoid air cells may increase the risk of ear disorders in patients with ET obstruction.

Grau *et al.*^20^ analyzed 22 patients with NPC following head and neck 2D-CRT and showed the radiation dose to the cochlea had a significant association with hearing loss. In contrast, Kwong *et al.*^21^ examined patients with NPC treated with RT and cisplatin, and reported that the radiation dose to the cochlea had no significant effect on ear disorders. Notably, our results are consistent with of Kwong *et al.*^21^. The differences between our observations and the study by Grau *et al.*^20^ may partially be explained by the use of different radiation techniques. Most previous studies were based on 2D-CRT, for which detailed dose-volume parameters are not available. In addition, the dosimetric parameters for all components of the auditory apparatus may be associated with ear disorders to some degree. However, according to our data, the ET.D_30_ and M.D_0.5CC_ are most relevant to post-radiotherapy ear disorders.

### Dose limits of critical structures for ear disorders

Multivariate analysis showed that an ET.D_30_ ≥ 52.75 Gy and M.D_0.5CC_ ≥ 41.04 Gy were negative prognostic factors for Grade 2 ear disorders in patients with NPC treated with IMRT. The incidence of ear disorders was 17% (13/38) in patients with an ET.D_30_ <52.75 Gy and 38% (45/119) in patients with an ET.D_30_ ≥ 52.75 Gy. These findings indicate that ET radiation-induced injury plays an important role in the development of ear disorders. In addition to radiation toxicity, Hsin *et al.*^22^ analyzed the effects of a one-sided primary tumor on ear disorders after radiation in patients with NPC, and provided additional evidence that ET injuries caused by the primary tumor influence the occurrence of ear disorders. In this study, we did not perform a separate analysis of the impact of the primary tumor on ear disorders because of the design of our trial. Therefore, it is difficult to determine whether pretreatment ET injuries associated with the tumor or radiation-induced damage of the ET play a more major role in the occurrence of ear disorders. Further research is needed to explore this issue.

Wang *et al.*^23^ reported lower rates of radiation-induced ear disorders in patients with NPC treated with IMRT when the mean dose to the middle ear cavity (including mastoid and TC) was limited to 34 Gy. In the current study, we failed to show the mean doses to the mastoid and TC were relevant to the occurrence of ear disorders, but found that a M.D_0.5CC_ ≥ 41.04 Gy was significantly associated with Grade 2 ear disorders. The frequency of ear disorders was 21% (13/62) in the group with a M.D_0.5CC_ <41.04 Gy and 33% (45/145) in the group with a M.D_0.5CC_ ≥ 41.04 Gy. This inconsistency is probably due to the fact that the newly-identified predictors (i.e. M.D_0.5CC_) reported in the current study are associated with the mean dose to the middle ear cavity, but have a more significant impact on ear disorders.

## Conclusion

To our knowledge, this is the first single-institution study to analyze the association between dose–volume effects and radiation-induced ear disorders in patients with NPC treated with IMRT. Clinicians should recognize that patients are at an increased risk for ear disorders if the ET.D_30_ is >52.75 Gy and M.D_0.5CC_ is >41.04 Gy. This study provides valuable insight into risk factors for ear disorders, and will help to optimize NPC treatment planning to improve tumor control and avoid side effects.

## Additional Information

**How to cite this article**: Yao, J.-J. *et al.* Dose-volume factors associated with ear disorders following intensity modulated radiotherapy in nasopharyngeal carcinoma. *Sci. Rep.* **5**, 13525; doi: 10.1038/srep13525 (2015).

## Figures and Tables

**Table 1:** The distribution of clinicopathological features in the study population of 97 nasopharyngeal carcinoma patients treated with IMRT.

**Characteristic**	**No.**	**%**
Age, years		
<50	73	75.3
≥50	24	24.7
Gender		
Male	70	72.2
Female	27	27.8
Pathologic features		
WHO Type 1	1	1
WHO Type 2	96	99
T category∗		
T1	35	36.1
T2	54	55.7
T3	8	8.2
N category∗		
N0	36	37.1
N1	61	62.9
Stage group∗		
I	24	24.7
II	65	67.1
III	8	8.2

^∗^According to the 7th edition of the American Joint Committee on Cancer.

Abbreviations: IMRT, intensity-modulated radiotherapy; WHO, World Health Organization.

**Table 2:** Significant DVH parameters associated with Grade 2 ear disorders in univariate analysis.

**Variables**	**OR**	**OR (95% CI)**	***P***	**Variables**	**OR**	**OR (95% CI)**	***P***
ET.D_0.01cc_	5.22	1.74–19.26	0.006	Mastoid.V45	3.28	1.16–10.04	0.028
ET.D_0.02cc_	4.92	1.70–16.24	0.005	Mastoid.Gy.	4.85	1.50–21.68	0.016
ET.D_0.03cc_	4.97	1.66–18.35	0.007	IAC.0.01	4.22	1.41–15.55	0.016
ET.D_0.04cc_	5.09	1.70–18.80	0.006	IAC.0.02	4.32	1.44–15.91	0.014
ET.D_0.05cc_	5.48	1.82–20.23	0.004	IAC.0.03	4.32	1.44–15.91	0.014
ET.D_0.06cc_	5.34	1.78–19.74	0.005	IAC.0.04	4.03	1.34–14.84	0.020
ET.D1	4.74	1.58–17.49	0.009	IAC.0.05	4.12	1.37–15.19	0.018
ET.D10	4.74	1.58–17.49	0.009	IAC.0.06	4.03	1.34–14.84	0.020
ET.D20	4.52	1.51–16.68	0.012	IAC.D1	3.73	1.32–11.44	0.015
ET.D30	5.34	1.78–19.74	0.005	IAC.D10	3.68	1.23–13.54	0.029
ET.D35	4.32	1.44–15.91	0.014	IAC.D20	3.96	1.23–17.70	0.036
ET.D40	4.22	1.41–15.55	0.016	IAC.D30	4.14	1.28–18.52	0.031
ET.D5	4.74	1.58–17.49	0.009	IAC.D35	3.96	1.23–17.70	0.036
ET.D50	4.03	1.34–14.84	0.020	IAC.D40	3.96	1.23–17.70	0.036
ET.D60	3.82	1.33–12.59	0.017	IAC.D45	3.87	1.20–17.31	0.040
ET.V50	3.82	1.33–12.59	0.017	IAC.D5	3.59	1.20–13.24	0.032
ET.V55	4.52	1.51–16.68	0.012	IAC.D50	3.96	1.23–17.70	0.036
ET.V60	9.27	1.82–169.56	0.033	IAC.V45	4.05	1.25–18.11	0.033
Mastoid.D_0.5cc_	4.04	1.43–12.41	0.010	TC.D_0.01cc_	6.09	1.88–27.24	0.006
Mastoid.D_1cc_	3.93	1.39–12.07	0.012	TC.D_0.02cc_	4.22	1.41–15.55	0.016
Mastoid.D_2cc_	3.45	1.22–10.57	0.022	TC.D_0.05cc_	5.56	1.72–24.84	0.009
Mastoid.D_4cc_	4.74	1.47–21.20	0.018	TC.D_0.1cc_	3.94	1.31–14.51	0.022
Mastoid.D10	4.79	1.66–15.82	0.005	TC.Gy.	9.27	1.82–169.56	0.033
Mastoid.D20	6.09	1.88–27.24	0.006	TC.D1	4.96	1.54–22.17	0.015
Mastoid.D30	4.97	1.66–18.35	0.007	TC.D10	4.32	1.44–15.91	0.014
Mastoid.D35	4.74	1.47–21.20	0.018	TC.D20	9.05	1.77–165.57	0.035
Mastoid.D40	4.24	1.31–18.94	0.028	TC.D5	4.22	1.41–15.55	0.016
Mastoid.D5	5.33	1.84–17.62	0.003	TC.V40	9.72	1.90–177.76	0.029
Mastoid.V30	8.84	1.73–161.66	0.037	TC.V45	6.38	1.97–28.55	0.005
Mastoid.V40	5.19	1.79–17.14	0.003	TC.V50	9.95	1.95–181.97	0.028

Abbreviations: ET, Eustachian tube; IAC, internal auditory canal; TC, tympanic cavity;

D_0.1cc_, dose to 0.1 ml of the volume; D1, dose to 1% of the volume; V30, volume of 30% that received more than 30 Gy; Mastoid.Gy, mean dose to the mastoid; TC.Gy, mean dose to the tympanic cavity.

**Table 3:** ROC curve and multivariate analysis of DVH parameters significantly associated with Grade 2 ear disorders.

**Variables**	**AUC**	**95% CI**		Cutoffpoint(Gy)	**Sensitivity**	**Specificity**	**OR**	***P***
		**Lower**	**Upper**					
ET.D_30_	0.69	0.56	0.80	52.75	0.75	0.64	3.77	0.012
M.D_0.5CC_	0.65	0.51	0.77	41.04	0.63	0.71	1.27	0.033

Abbreviations: ROC, receiver operating characteristic; AUC, area under curve; OR, odd ratios; ET.D_30_, dose to 30% of the Eustachian tube volume; M.D_0.5CC_, dose to 0.5 ml of the mastoid volume.
